# Field Evaluation and Calibration of Low-Cost Air Pollution Sensors for Environmental Exposure Research

**DOI:** 10.3390/s22062381

**Published:** 2022-03-19

**Authors:** Jianwei Huang, Mei-Po Kwan, Jiannan Cai, Wanying Song, Changda Yu, Zihan Kan, Steve Hung-Lam Yim

**Affiliations:** 1 Institute of Space and Earth Information Science, The Chinese University of Hong Kong, Hong Kong, China; jianwei.huang@link.cuhk.edu.hk (J.H.); jncai@cuhk.edu.hk (J.C.); wysong@link.cuhk.edu.hk (W.S.); yuchangda@link.cuhk.edu.hk (C.Y.); zihankan@cuhk.edu.hk (Z.K.); 2 Department of Geography and Resource Management, The Chinese University of Hong Kong, Hong Kong, China; 3 Asian School of the Environment, Nanyang Technological University, Singapore 639798, Singapore; steve.yim@ntu.edu.sg; 4 Lee Kong Chian School of Medicine, Nanyang Technological University, Singapore 639798, Singapore; 5 Earth Observatory of Singapore, Nanyang Technological University, Singapore 639798, Singapore

**Keywords:** particulate matter, AirBeam2, low-cost sensors, urban environments, different aggregated temporal units, sensor calibration

## Abstract

This paper seeks to evaluate and calibrate data collected by low-cost particulate matter (PM) sensors in different environments and using different aggregated temporal units (i.e., 5-s, 1-min, 10-min, 30 min intervals). We first collected PM concentrations (i.e., PM_1_, PM_2.5_, and PM_10_) data in five different environments (i.e., indoor and outdoor of an office building, a train platform and lobby of a subway station, and a seaside location) in Hong Kong, using five AirBeam2 sensors as the low-cost sensors and a TSI DustTrak DRX Aerosol Monitor 8533 as the reference sensor. By comparing the collected PM concentrations, we found high linearity and correlation between the data reported by the AirBeam2 sensors in different environments. Furthermore, the results suggest that the accuracy and bias of the PM data reported by the AirBeam2 sensors are affected by rainy weather and environments with high humidity and a high level of hygroscopic salts (i.e., a seaside location). In addition, increasing the aggregation level of the temporal units (i.e., from 5-s to 30 min intervals) increases the correlation between the PM concentrations obtained by the AirBeam2 sensors, while it does not significantly improve the accuracy and bias of the data. Lastly, our results indicate that using a machine learning model (i.e., random forest) for the calibration of PM concentrations collected on sunny days generates better results than those obtained with multiple linear models. These findings have important implications for researchers when designing environmental exposure studies based on low-cost PM sensors.

## 1. Introduction

Much research on the health impacts of individual exposure to particulate matter (e.g., PM_1_, PM_2.5_, and PM_10_) is based on people’s residential neighborhoods [1,2]. However, using people’s residential neighborhoods for environmental health research can lead to the uncertain geographic context problem (UGCoP) and the neighborhood effect averaging problem (NEAP) [3,4,5,6,7,8,9]. The UGCoP stresses that using different geographically delineated contextual areas could lead to different research findings about the health effects of environmental factors [10]; and the NEAP suggests that ignoring people’s daily mobility and exposure to nonresidential contexts could lead to biased estimations of personal exposure, specifically, the exposure of people who have very high or low residence-based exposures would tend toward the average of the population when their daily mobility is taken into account (i.e., mobility-based exposures) [11,12].

To address these methodological issues, some recent studies have applied a mobility-based exposure approach to simultaneously capture the spatiotemporal variability of PM concentrations and human mobility [13,14,15]. The mobility-based exposure approach uses low-cost sensors to measure individual-level PM-concentration exposure in different types of environments, which is different from conventional environmental exposure studies (e.g., using residential neighborhoods). Furthermore, the mobility-based exposure approach has advanced the acquisition of accurate data on human space-time behaviors considerably in different types of environments (e.g., workplaces, supermarkets, and transport stations). It provides detailed space–time information that is essential for a personal environmental exposure assessment and has been increasingly used in health research [16,17,18]. The mobility-based exposure approach requires people to carry a portable low-cost PM sensor and a GPS sensor, which can simultaneously monitor individuals’ geographic locations and measure the real-time PM concentrations in people’s immediate surroundings at very fine spatiotemporal resolutions [19]. Specifically, a mobility-based exposure approach can capture individuals’ real-time location (i.e., longitude and latitude) and exposure to PM concentrations every second [4]. Although such an approach can significantly improve the accuracy of people’s PM exposure assessment compared with conventional methods (e.g., using residential neighborhoods), our knowledge on the reliability of data collected by low-cost PM sensors in different types of environments is still very limited to date.

Some studies have found that the data collected by low-cost PM sensors are not as accurate as those collected by conventional monitoring stations [20,21,22]. Specifically, various studies and reports have indicated that certain ambient physical conditions (e.g., temperature, humidity, and precipitation) can significantly affect the performance of low-cost PM sensors [23,24]. For instance, by comparing the measurements of low-cost air quality sensors (i.e., Airbeam and the Alphasense Optical Particle Counter) with the measurements of high-end instruments (i.e., the GRIMM 11-R optical particle counter and the Met One beta attenuation monitor), Mukherjee et al. [20] found that sensor measurements were influenced by the meteorological conditions and the distribution of aerosol size. Sousan et al. [25] conducted simulation experiments in the laboratory and suggested that the reliability of data provided by low-cost PM sensors can be improved if calibrated differently for various environmental conditions (e.g., different environmental and occupational settings) using site-specific calibration factors.

Although previous studies provided a useful foundation for improving the accuracy of data recorded by low-cost portable PM sensors, they did not examine how the performance of these sensors varies in different types of environments for people’s daily life. Specifically, the performance of low-cost PM sensors may vary between different types of environments (e.g., workplaces, supermarkets, and transport stations) due to different environmental conditions (e.g., temperature, humidity, and precipitation). Note that most people are exposed to different types of environments in their daily life, since they may travel around and visit different types of places and venues. Thus, low-cost PM sensors may have different levels of performance when people are required to carry the sensors while conducting their daily activities in mobility-based environmental exposure studies. Besides, although the mobility-based exposure approach can measure individuals’ PM concentrations every second, the accuracy of the data recorded by low-cost PM sensors may vary due to different aggregation levels of the temporal unit (i.e., 1 s, 1 min, 10 min, etc.). Thus, it is important to evaluate and calibrate the data recorded by low-cost PM sensors under different types of environments using different aggregated temporal units for mobility-based environmental exposure studies. The contribution of such an analysis is two-fold. On one hand, the new knowledge generated can help in the development and enhancement of the effectiveness of a research design for mobility-based environmental exposure studies. On the other hand, the uncertainties arising from the performance of low-cost PM sensors can be examined and addressed using appropriate calibration models.

Motivated by the abovementioned research gaps, this study seeks to evaluate and calibrate low-cost PM sensors in different types of urban environments using different aggregated temporal units. Specifically, we first used five AirBeam2 low-cost sensors and a TSI DustTrak DRX Aerosol Monitor 8533 as a reference sensor to collect PM concentrations (i.e., PM_1_, PM_2.5_, and PM_10_) data in five types of environments (i.e., an indoor location and an outdoor location of an office building, the train platform and lobby of a subway station, and a seaside location) in Hong Kong. Then, using the PM concentrations data aggregated into 5-s, 1-min, 10-min, and 30 min intervals, the performance of the AirBeam2 sensors was evaluated based on the correlation, accuracy, and bias of the data collected in different environments. Furthermore, we calibrated the 1 min average PM concentrations data using the temperature and humidity data recorded by the AirBeam2 sensors based on multiple linear regression (MLR) and random forest (RF) (a machine learning method). Finally, we discuss how different types of environments and different aggregated temporal units would affect the performance of low-cost PM sensors and their implications for better design in mobility-based environmental exposure studies.

## 2. Materials and Methods

### 2.1. Sensors and Study Sites

The low-cost PM sensors used in this study are five AirBeam2 sensors (HabitatMap, Brooklyn, NY, USA). We used AirBeam2 sensors because they are lightweight (198.5 g), have a long battery life (10 h when fully charged), and are widely used in mobility-based environmental exposure studies [4,5,19,26]. Furthermore, AirBeam2 sensors can measure fine particulate matter (PM_1_, PM_2.5_ and PM_10_), temperature, and relative humidity. Note that the PM concentrations measured by AirBeam2 sensors have been calibrated based on equations developed by fitting the data to the GRIMM EDM180. Past studies have evaluated the performance of AirBeam2 sensors by comparing them with different reference instruments. For instance, by comparing AirBeam2 measurements to measurements by a TSI DustTrak DRX Aerosol Monitor 8533 in a concentrated air pollutants (CAPS) chamber, Michael and Lim [27] found that the PM concentrations measured by AirBeam2 have a highly linear relationship with the data recorded by the DustTrak sensor (i.e., R^2^ = 0.89 for PM_2.5_ and R^2^ = 0.88 for PM_1_). In field tests performed by different researchers, the measurements of PM_2.5_ of AirBeam sensors strongly correlated with the measurements obtained by a GRIMM monitor (i.e., R^2^ = 0.80–0.99) [20]. While these studies provided significant evidence to demonstrate the reliability of AirBeam2 sensors in particulate matter (PM) measurements, they did not examine how the performance of the AirBeam2 sensors vary between different types of urban environments (e.g., Mass Transit Railway [MTR] stations, supermarkets, seaside, and offices). Thus, this paper seeks to evaluate the performances of AirBeam2 sensors in different types of urban environments.

To evaluate the accuracy and bias of the AirBeam2 sensors, we compared the PM concentrations they recorded with the data obtained by a TSI DustTrak DRX Aerosol Monitor 8533 sensor. The DustTrak sensor offers simultaneous measurements of PM concentrations for different particle sizes (PM_1_, PM_2.5_, PM_3_, PM_10_, and total particles) [28]. The DustTrak is widely used in measuring PM concentrations in indoor and outdoor environments and evaluating low-cost PM sensors [29,30,31,32,33,34].

The AirBeam2 and DustTrak sensors were employed in this study to measure PM concentrations in different types of environments in Hong Kong. The city has a highly transit-oriented development (TOD) around Victoria Harbour due to limited land resources, and more than 90% of the people in Hong Kong are serviced by the public transport system [35,36]. Note that the yearly average PM_2.5_ and PM_10_ concentrations in Hong Kong are 16.7 μg/m^3^ and 29.7 μg/m^3^ according to the data collected by the Hong Kong Environmental Protection Department, covering the period from October 2020 to September 2021 [37]. In addition, Hong Kong has a subtropical climate. Its 2021 monthly average temperatures range from 16.2 °C (January) to 29.7 °C (July) [38]. Thus, people in Hong Kong usually conduct their daily activities in places or venues around Mass Transit Railway (MTR) stations or seaside locations (e.g., walking, running, or taking a ferry). Note that the MTR stations in Hong Kong usually have a lobby with several shops (e.g., cake shops, banks, coffee shops, and newsstands), station facilities (e.g., customer service centers, restrooms or toilets, and breastfeeding areas), and platforms for passengers to take the train.

We selected three locations with five types of environments in Hong Kong to collect the PM concentrations data. Table 1 provides a brief description of the selected environments and the date of data collection, including the indoor and outdoor areas of an office building, the platform and the lobby of an MTR station, and a seaside location near a ferry pier. The office is in the Institute of Space and Earth Information Science at The Chinese University of Hong Kong. The MTR station and ferry pier are in Hung Hom, which is a major transport hub in Hong Kong with an MTR station, a ferries pier, a cross-harbor tunnel, and a terminus of cross-border bus services with transport to major cities in Mainland China. Note that the day we collected data in the office (outdoor) was rainy, and the days we collected data in other environments were all sunny. The patterns of people’s daily mobility in a city tend to be quite regular over days (e.g., weekdays and weekends) [36,39,40]. Thus, mobility-based environmental exposure studies usually require people to carry an AirBeam2 sensor for two days (one weekday and one weekend) [4,5,19,26,41]. Hence, for each type of environment, we collected data from 9:00 to 17:00. For PM concentrations data in the office (indoor), data were missing for one of the AirBeam2 sensors from 12:30 to 13:00. Besides, Table 1 also presents the average PM_2.5_ and PM_10_ concentrations reported by the Hong Kong Environmental Protection Department on the days of our data collection (i.e., Sha Tin, Sham Shui Po, Mong Kok, and Kwun Kong monitoring stations) [37].

### 2.2. Evaluation of Correlation, Accuracy, and Bias of Data Collected with Low-Cost PM Sensors

Data collected from the Airbeam2 sensors were time-paired with the data collected by the DustTrak sensor every second using Python. First, we used scatterplots and descriptive statistics (i.e., the mean and standard deviation) for the 1 min average concentrations of PM_1_, PM_2.5_, and PM_10_ recorded by the AirBeam2 and DustTrak sensors to explore the general patterns of the data. Then, using the 1 min average PM concentrations recorded by the sensors, the data of the AirBeam2 sensors were examined based on their correlation, accuracy, and bias in different types of environments. Specifically, the Pearson coefficient was used to explore the correlation between the data recorded by the five AirBeam2 sensors. A linear regression model was used to measure the accuracy (i.e., the slope and R^2^) of the AirBeam2 sensors compared to the DustTrak sensor. Finally, the bias (how well the Airbeam2 data agreed with the DustTrak data) was evaluated for the 1 min average PM concentrations using the following Equation (1):

(1) $$B_{1s}=\frac{1}{n}\sum \frac{y_{i}-x_{i}}{x_{i}}$$

where y is the observed PM concentrations of the AirBeam2 sensors, x is the observed PM concentrations of the DustTrak sensor, i is the data pair index, and n is the total number of data pairs. In addition, we also aggregated the collected PM concentrations data into 5 s, 1 min, 10 min, and 30 min intervals to explore how the correlation, accuracy, and bias of the data recorded by the AirBeam2 sensors would be affected due to the use of different aggregated temporal units.

### 2.3. Machine Learning-Based Calibration Model Development and Validation

In this subsection, we focus on developing and validating calibration models based on the temperature and humidity data recorded by the AirBeam2 sensors using multiple linear regression (MLR) and the random forest (RF) method. Equation (2) was used in the MLR model.

(2) $$Y=\beta_{1}X+\beta_{2}Temp+\beta_{3}RH+\beta_{0},$$

where *Y* is the PM concentrations (i.e., PM_1_, PM_2.5_, and PM_10_) recorded by the DustTrak sensor, *X* is PM concentrations recorded by the AirBeam2 sensors, *Temp* is the temperature reported by the AirBeam2 sensors, and *RH* is the relative humidity reported by the AirBeam2 sensors. Note that all data collected in the selected environments were used and aggregated into 1 min intervals in the model.

We also used the same data to develop a calibration model based on RF, which is a decision tree-based machine learning method widely used for classification or regression [42,43,44]. In the RF method, a collection of regression or classification trees is first drawn from different bootstrap samples of the training data. Then, each tree acts as a regression or classification function on its own, and the final output is taken as the majority vote for classification or average of the individual tree for regression. In this study, the RF model was applied by using scikit-learn library in Python, and the parameters for the model were selected by using an optimizing hyperparameter tuning method [45].

For the MLR and RF models, the ten-fold cross-validation method was applied to fit better models. For the ten-fold cross-validation method, each model was trained using 80% of the data, and the remaining 20% of the data were used to validate the model. This process was repeated ten times and all the data were used to validate the calibrated results. In addition, the performance of the calibration models was evaluated by comparing data between model-calibrated PM concentrations data and the data recorded by the DustTrak sensor using R^2^, bias (i.e., Formula (1)), mean error (ME) (µg/m^3^) and root mean squared error (RMSE) (µg/m^3^).

(3) $$\mathrm{ME}\;=\frac{1}{n}\overset{n}{\underset{i=1}{\sum }}|y_{i}-x_{i}|,$$

(4) $$\mathrm{RMSE}\;=\sqrt{\frac{\int_{i=1}^{n}\left(y_{i}-x_{i}\right)}{n}},$$

where *n* is the number of data pairs, $y_{i}$ is the calibrated PM concentrations generated by the MLR and RF models, and $x_{i}$ is the PM concentrations recorded by the DustTrak sensor.

Recall that the day we collected data in the outdoor area of an office building was a rainy day, and all the other days were sunny. To further explore how the performance of the calibration models would be affected by weather conditions, we also excluded the data collected in the office (outdoor) environment and reanalyzed the MLR and RF models. The training data and validating data were randomly chosen from the data set using a method similar to that described above.

## 3. Results

### 3.1. PM_1_, PM_2.5_, and PM_10_ Concentrations Collected by Sensors in Different Environments

**Figure 1:**
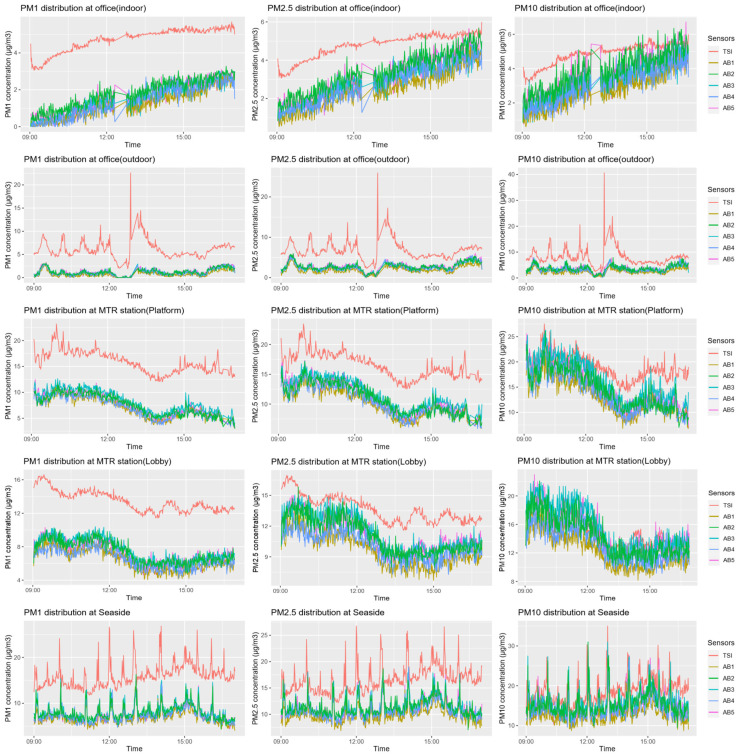
The 1 min PM1, PM2.5, and PM10 average concentrations (µg/m^3^) reported by AirBeam2 and DustTrak sensors in different environments.

In this subsection, we first use scatterplots and descriptive statistics (i.e., the mean and standard deviation) for the 1 min average PM concentrations recorded by the AirBeam2 and DustTrak sensors to explore the general patterns of the data. Figure 1 presents the distribution of the 1 min PM_1_, PM_2.5_, and PM_10_ average concentrations reported by the AirBeam2 and DustTrak sensors in the indoor and outdoor space of an office building, the platform and the lobby of an MTR station, and a seaside location. The results indicate that the PM concentrations recorded by the DustTrak sensor are generally higher than those recorded by the AirBeam2 sensors in different environments, which is in line with the results of previous studies [27]. Specifically, the 95% confidence interval on the mean of the differences between the PM concentrations reported by the AirBeam2 and DustTrak sensors range from 1.54 to 11.04 for PM_1_, −0.01 to 8.17 for PM_2.5_, and −2.41 to 8.47 for PM_10_.

Table 2 presents the descriptive statistics of the 1 min PM_1_, PM_2.5_, and PM_10_ average concentrations obtained by the AirBeam2 and DustTrak sensors in different environments. The results indicate that the mean values of the PM concentrations reported by the AirBeam2 sensors are lower than that of the DustTrak sensor, while the differences between the mean values reported by the sensors decrease as the size of PM increases. In other words, the PM_10_ concentrations recorded by the AirBeam2 and DustTrak sensors have the smallest difference, while the PM_1_ concentrations present the largest difference. These results are consistent with our earlier findings from Figure 1. In addition, the results also indicate that the mean values and standard deviations of PM concentrations recorded by the AirBeam2 sensors under different environments are generally similar to each other. For instance, the mean values of the PM concentrations recorded by the AirBeam2 sensors in the office (indoor) range from 1.21 to 1.81 for PM_1_, 2.47 to 3.36 for PM_2.5_, and 2.74 to 3.84 to PM_10_, while the standard deviations range from 0.76 to 0.84 for PM_1_, 0.98 to 1.04 for PM_2.5_ and 1.01 to 1.13 for PM_10_. The mean values and standard deviations of the PM concentrations recorded by the AirBeam2 sensors in other types of environments present a similar patterns to that of the office (indoor).

By comparing the mean values of PM concentrations reported by the AirBeam2 sensors and the monitoring stations, we found that the mean values of the PM_2.5_ and PM_10_ concentrations reported by the AirBeam2 sensors were generally lower than that of the PM concentrations reported by the monitoring stations (see Table 1). Furthermore, by comparing the mean values of the PM concentrations reported by the DustTrak sensor and the monitoring sensors in the selected environments (except the office [indoor]), we found that the mean values of the PM_2.5_ concentrations reported by the DustTrak sensor are generally similar to that of the PM_2.5_ concentrations reported by the monitoring stations, while the mean values of the PM_10_ concentrations reported by the DustTrak sensor are lower than that of the PM_10_ concentrations reported by the monitoring stations. One of the potential reasons is that the DustTrak sensors may underestimate PM_10_ concentration [46]. Meanwhile, the mean values of the PM_2.5_ and PM_10_ concentrations in the office (indoor) environment reported by the DustTrak sensor are lower than that of the PM_2.5_ and PM_10_ concentrations reported by the monitoring stations. The potential reasons include that there are air filters and air cleaning devices in the office (indoor). Using these devices and closing the windows improves the air quality in the office (indoor) compared that in the other office (outdoor).

### 3.2. Sensor Performance in Different Environments

**Figure 2:**
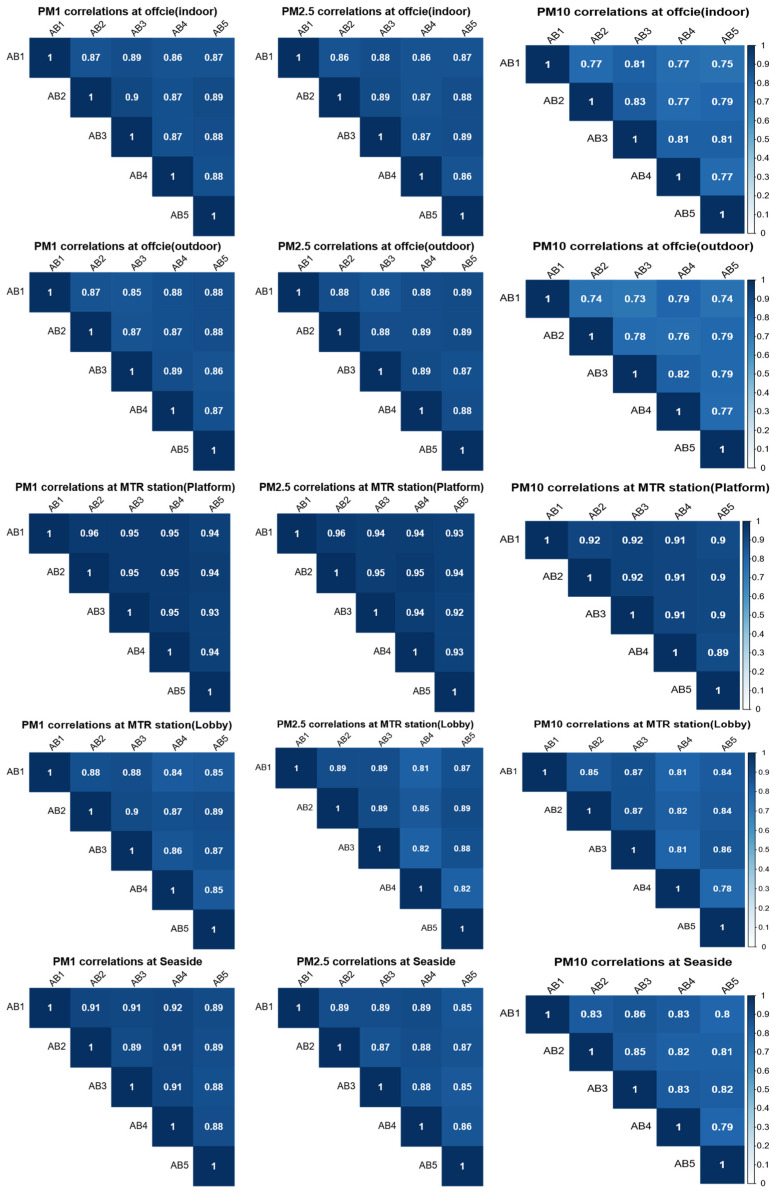
Correlation matrix for the 1 min PM_1_, PM_2.5_, and PM_10_ average concentrations (µg/m^3^) reported by the AirBeam2 sensors.

In this subsection, the data collected by the AirBeam2 sensors in different environments are evaluated by their correlation, accuracy, and bias using the 1 min average PM concentrations recorded by the AirBeam2 and DustTrak sensors. Specifically, the Pearson coefficient was used to explore the correlation between the data reported by the five AirBeam2 sensors. Figure 2 presents the correlations between the 1 min PM_1_, PM_2.5_, and PM_10_ average concentrations obtained by the AirBeam2 sensors in different environments. First, the correlation coefficients range from 0.73 to 0.96, which indicates a high linearity and correlation between the PM concentrations recorded by the AirBeam2 sensors in all the selected environments. Then, we found that the correlation coefficients for data collected in the MTR station (platform and lobby) are higher than those collected in other environments. Specifically, data obtained by the AirBeam2 sensors on the platform of the MTR station have the highest correlation coefficients for the 1 min PM_1_, PM_2.5_, and PM_10_ average concentrations. In addition, the results also indicate that the correlation coefficient decreases as the size of PM increases. In other words, the correlation coefficient for the 1 min PM_1_ average concentrations has the highest value, while it has the smallest value for the 1 min PM_10_ average concentrations.

Besides, the linear regression model and bias assessment (i.e., Formula (1)) were used to measure the accuracy of the AirBeam2 sensors compared to the DustTrak sensor. Table 3 shows the results of the evaluation for different environments. First, we found that the 1 min PM average concentrations recorded by the AirBeam2 sensors in the office (indoor) and MTR station (platform and lobby) environments, in general, have a linear relationship with the data reported by the DustTrak sensor (i.e., R^2^ values range from 0.61 to 0.78, and slope values range from 0.44 to 0.95). The 1 min PM average concentrations obtained by the AirBeam2 sensors in the office (outdoor) and seaside environments have a non-linear relationship with the data recorded by the DustTrak sensor (i.e., R^2^ values range from 0.11 to 0.23). Note that we also found that the R^2^ values range from 0.05 to 0.11 for PM_1_, 0.05 to 0.33 for PM_2.5_, and 0.23 to 0.58 for PM_10_ by using a high-order curve (second-order and third-order) to fit the data obtained in the office (outdoor) and seaside environments. These results are different from those obtained in previous studies, which found a highly linear relationship (R^2^ = 0.88–0.89) between PM concentrations recorded by AirBeam2 sensors and a DustTrak sensor [27]. A possible reason for this is that AirBeam2 sensors are significantly affected in a relatively high humidity environment with hygroscopic salts [24,47,48]. Specifically, the sensitivity of AirBeam2 sensors may be affected due to the fog droplets, which may be detected as particles in a relatively high humidity environment [49]. Because a seaside location is a relatively high humidity environment with a high level of hygroscopic salts, the PM concentrations reported by the AirBeam2 sensors in the seaside location in this study would have lower accuracy than the PM concentrations collected in other environments. In addition, the relative humidity of the atmosphere increases during rainy weather, often approaching 100%. Thus, the PM concentrations reported by the AirBeam2 sensors on a rainy day would be significantly affected and have lower accuracy than the PM concentrations collected in other environments.

In addition, the bias percentage between the data recorded by the AirBeam2 sensors and the data collected by the DustTrak sensor decreases as the size of PM increases. This result is in line with the results reported earlier in Section 3.1. The differences between the mean values of PM concentrations recorded by the sensors decreases as the size of PM increases.

### 3.3. Sensor Performance in Different Temporal Units

In this subsection, we focus on exploring how the correlation, accuracy, and bias of PM concentrations recorded by the AirBeam2 sensors are affected by the use of different aggregated temporal units. Figure S1 presents the correlation between the PM_1_, PM_2.5_, and PM_10_ concentrations reported by the AirBeam2 sensors in different environments and different temporal units (i.e., 5 s, 1 min, 10 min, and 30 min). We found that the correlation increases as the aggregation level of the temporal unit increases for all environments. Specifically, the correlation coefficients are around 0.6 to 0.7 when the temporal unit is 5 s, while the correlation coefficients range from 0.95 to 1 when the temporal unit is 30 min. In addition, the correlation coefficients for different temporal units are not affected by changes in PM size.

Figure S2 presents the results of the multiple linear regression models for the data recorded by the AirBeam2 and DustTrak sensors in different environments and different temporal units. The result indicates that the R^2^ values generally increase as the aggregation level of the temporal units increases (i.e., from 5 s to 30 min) in the office (indoor) and MTR station (platform and lobby) environments. For the office (outdoor) environment, increasing the aggregation level of the temporal units (i.e., from 5 s to 30 min) does not significantly affect the results of the multiple linear regression between the PM concentrations reported by the AirBeam2 and DustTrak sensors. For the seaside environment, the R^2^ values first increase as the aggregation level of the temporal units increases (i.e., from 5 s to 10 min) and then decrease for the 30 min interval. Thus, the R^2^ values reach the maximum value when the temporal unit is 10 min.

Figure S3 presents the results of the bias percentage between the PM concentrations recorded by the AirBeam2 and DustTrak sensors in different environments and different temporal units. The results suggest that changing the aggregated temporal units does not significantly reduce the bias. Specifically, the bias values may increase or decrease with an increase in the aggregation level of the temporal units (i.e., from 5 s to 30 min) in different environments. These results suggest that increasing the aggregation level of the temporal units increases the correlation of the PM concentrations obtained by the AirBeam2 sensors in different environments, while it does not significantly improve the accuracy and bias for the data.

### 3.4. Machine Learning-Based Calibration and Validation

In this subsection, we focus on developing and validating calibration models that are based on the temperature and humidity data reported by the AirBeam2 sensors using multiple linear regression (MLR) and the random forest (RF) method. We first used all PM concentrations collected in the selected environments and aggregated them into 1 min intervals. Table 4 presents the results of the calibrated models based on the data collected in all selected environments. The results indicate low-to-moderate linearity (i.e., R^2^ values range from 0.16 to 0.59) between the calibrated 1 min PM_1_, PM_2.5_ and PM_10_ average concentrations recorded by the AirBeam2 and DustTrak sensors based on the MLR and RF models. Furthermore, the results also suggest that the R^2^ values between the calibrated PM concentrations data recorded by the AirBeam2 sensors and those recorded by the DustTrak sensor decrease as the size of PM increases. In addition, the bias percentage significantly decreases (i.e., the bias percentage range from −2.54 to 1.23) after the PM concentrations data were calibrated.

Besides, we also excluded data collected in the office (outdoor) environment and rerun the MLR and RF models. Table 5 presents the results of the calibrated models based on the PM concentrations collected in the office (indoor), MTR station (platform and lobby), and the seaside location. First, the results indicate a high linearity (i.e., R^2^ values range from 0.89 to 0.95) between the calibrated 1 min PM_1_, PM_2.5_, and PM_10_ average concentrations recorded by the AirBeam2 and DustTrak sensors based on the MLR and RF models. Furthermore, the results also suggest that R^2^ values between the calibrated PM concentrations recorded by the AirBeam2 and DustTrak sensors are not affected by the size of PM. In addition, the bias percentage significantly decreased (i.e., the bias percentage ranges from −1.21 to −0.04) after the data were calibrated.

Additionally, by comparing the results of cross-validation ME, RMSE, and bias percentage between the MLR and RF models in Table 4 and Table 5, we found that the RF models can generate more robust calibrated PM concentrations for the AirBeam2 sensors than the MLR models, as the values of the ME, RMSE and bias percentage reported by the RF models are generally smaller than that those reported by the MLR models.

These results are consistent with previous findings [24]. The results imply that there is a non-linear relationship between the temperature, relative humidity, and PM concentrations reported by AirBeam2 sensors during rainy weather. It is thus difficult to derive any appropriate correction models.

## 4. Discussion

Previous studies have focused on establishing different linear models to calibrate PM concentrations recorded by low-cost portable sensors in certain environments with different concentration levels based on the applications in the literature [50,51]. This study is important because it extends previous studies by presenting how the correlation, accuracy, and bias of PM concentrations reported by low-cost PM sensors (i.e., AirBeam2 sensors) would be affected by different types of urban environments and different aggregated temporal units. Specifically, the results reveal that the PM (i.e., PM_1_, PM_2.5_, and PM_10_) concentrations recorded by the AirBeam2 sensors are generally lower than those obtained by the monitoring stations and the DustTrak sensor in different environments. The results show the high linearity and correlation between the data recorded by the AirBeam2 sensors in different environments for the three types of PM concentrations. By comparing the data collected by the AirBeam2 and DustTrak sensors, the results also indicate that the accuracy and bias of the data recorded by the AirBeam2 sensors are significantly affected by weather conditions (i.e., rainy day) and environments with a relatively high humidity and a high level of hygroscopic salts (i.e., seaside). Meanwhile, the correlation, accuracy, and bias of the PM concentrations recorded by the AirBeam2 sensors are affected by PM size. In addition, by using data aggregated in different temporal units (i.e., 5 s, 1 min, 10 min, and 30 min), the results suggest that increasing the aggregation level of the temporal units (i.e., from 5 s to 30 min) significantly increases the correlation coefficients for PM concentrations recorded by the AirBeam2 sensors in different environments, while it does not significantly improve the accuracy and bias of the data. Lastly, the calibration models indicate that using random forest (RF) models would generate better results than multiple linear regression (MLR) models for the data collected on sunny days (i.e., excluding the data collected on rainy days). The findings have several important implications for researchers when designing mobility-based environmental exposure studies that use low-cost PM sensors.

First, our results reveal a high linearity and correlation between the data recorded by the AirBeam2 sensors in different environments when the aggregated temporal unit is larger than a 5-s interval (e.g., 1 min). Specifically, the correlation coefficients between the PM concentrations reported by the AirBeam2 sensors are not significantly affected by the environment and the weather conditions (e.g., a relatively high humidity environment with a high level of hygroscopic salts or a rainy day). Thus, our findings suggest that researchers should perform tests to detect how different aggregated temporal units may affect the results when used PM concentrations data reported by low-cost PM sensors for mobility-based environmental exposure studies, which largely compare individual exposure data. For instance, by comparing the PM concentrations recorded by low-cost PM sensors with certain aggregated temporal units (e.g., 1-min), studies can explore which social groups have a disadvantage in exposure to air pollution and thus shed light on environmental inequality issues.

Second, our results indicate that the accuracy and bias of PM concentrations recorded by the AirBeam2 would be affected by weather conditions (i.e., rainy days) and environments with relatively high humidity and a high level of hygroscopic salts (i.e., seaside). Thus, when studying the health effects of PM exposure, low-cost PM sensors, without being properly calibrated, may generate misleading measurements. In addition, the results also highlight the importance of deriving calibration models based on machine learning methods (e.g., random forest) with consideration of weather conditions. Specifically, the results suggest that excluding data collected on rainy days based on random forest models for different environments can generate better outcomes for data calibration. Thus, researchers could develop machine learning models for data calibration in different environments when using low-cost PM sensors to explore how PM concentrations may affect people’s health. In addition, it should be kept in mind that weather conditions (e.g., rainy days) would significantly affect the accuracy and bias of the data collected by low-cost PM sensors.

However, the study has several limitations. First, due to the sensors’ (i.e., the AirBeam2 and DustTrak sensors) limitations, we developed and validated the calibration models based on the temperature and humidity data recorded by the AirBeam2 but could not consider other meteorological factors (e.g., wind speed) in different types of environments (e.g., outdoor and indoor environments of the office). Studies have shown that these factors may also be important in terms of affecting PM concentrations [52]. Thus, it is necessary to explore calibration models for PM concentrations data recorded by low-cost PM sensors in different environments, with consideration of other meteorological factors (e.g., wind speed) in future work.

Second, there is great potential to further extend our study on the evaluation and calibration of low-cost PM sensors to different seasons or weather conditions based on longer periods of data collection (e.g., including a weekday and a weekend day because people’s daily activities would be significantly different between weekdays and weekends). In addition, PM concentrations vary between different seasons or weather conditions. Thus, future studies should also consider how the data recorded by low-cost PM sensors may be affected in different seasons (e.g., winter and summer) or weather conditions (e.g., a snowy, dry, and rainy day).

Third, there is an issue with the performance of the DustTrak sensor in the PM_10_ concentration data collection. The DustTrak sensor may underestimate PM_10_ concentrations, and PM concentrations measured with the sensor may suffer from some random jumps (see Section 3.1). This bias could introduce uncertainties in the accuracy assessment of calibrated PM concentrations data for low-cost PM sensors. However, the methods applied in this study can still be used for designing better mobility-based environmental exposure studies that use low-cost PM sensors. Future studies would, of course, benefit from using more reliable portable PM concentrations sensors to capture more accurate data for the calibration of low-cost sensors in different types of indoor and outdoor environments (e.g., kitchen, smoking room, and various activity venues).

## 5. Conclusions

Low-cost PM sensor evaluation and calibration are crucial for establishing reliable PM concentrations measurements for mobility-based environmental exposure studies. Using data collected by five AirBeam2 sensors and one TSI DustTrak DRX Aerosol Monitor 8533 sensor in different environments (i.e., office [indoor and outdoor], MTR station [platform and lobby], and seaside) in Hong Kong, this study first assessed the reliability of the PM concentrations recorded by the AirBeam2 sensors during a 1 min average aggregation using correlation, accuracy, and bias analysis. Then, the study further explored how the correlation, accuracy, and bias of the PM concentrations recorded by the sensors are affected by the use of different temporal units (i.e., 5 s, 1 min, 10 min, and 30 min). Lastly, the study calibrated the data obtained by the sensors using multiple linear regression (MLR) and random forest (RF). The results suggest that the accuracy and bias of PM concentrations recorded by the AirBeam2 would be affected due to weather conditions (i.e., rainy days), environments with relatively high humidity, a high level of hygroscopic salts (i.e., seaside), and different aggregation levels of the temporal unit. The results also indicate that using RF models would generate better results than MLR models for the data collected on sunny days (i.e., excluding the data collected on rainy days). These results provide valuable insights (e.g., the selection of predictive variables) for the research community when designing environmental exposure studies that use low-cost PM sensors. First, it is necessary to perform tests to detect how different aggregated temporal units may affect the results of PM concentrations data reported by low-cost PM sensors. Second, using machine learning models (i.e., RF) for data calibration under different environments can generate better results than MLR models. In addition, more efforts are needed to develop calibration models under different weather conditions to obtain accurate PM concentrations data from low-cost PM sensors.

## Figures and Tables

**Table 1:** The selected environments and dates for data collection.

Urban Environments	Location with Latitude and Longitude	Data Collection Date	PM_2.5_ (μg/m^3^)	PM_10_ (μg/m^3^)
Office (Indoor)	Institute of Space and Earth Information Science, The Chinese University of Hong Kong (22.4213° N, 114.2068° E)	31 July 2021	9.8	16.7
Office (Outdoor) *		3 August 2021	4.6	7.8
MTR station (Platform)	Hung Hom Station (22.3034° N, 114.1814° E)	5 October 2021	13.5	34.3
MTR station (Lobby)		18 October 2021	14.5	23.2
Seaside	Hung Hom Ferry Pier (22.3011° N, 114.1902° E)	6 October 2021	14.7	36.5

* Note that 3 August 2021 is a rainy day.

**Table 2:** Statistic description of the 1 min PM_1_, PM_2.5_, and PM_10_ average concentrations (µg/m^3^) reported by AirBeam2 and DustTrak sensors in different environments.

PM_1_ Concentration (µg/m^3^)										
	Office (Indoor)		Office (Outdoor)		MTR Station (Platform)		MTR Station (Lobby)		Seaside	
Sensors	Mean	S.D.	Mean	S.D.	Mean	S.D.	Mean	S.D.	Mean	S.D.
DustTrak	4.71	0.65	6.02	2.02	15.88	2.09	13.46	1.23	15.89	2.51
AB1	1.56	0.81	1.25	0.78	7.71	2.17	7.29	1.32	7.52	1.68
AB2	1.45	0.79	1.09	0.69	8.33	2.11	7.59	1.38	8.01	1.78
AB3	1.21	0.78	0.75	0.62	6.98	1.94	6.39	1.27	6.88	1.53
AB4	1.41	0.84	1.23	0.76	7.04	1.96	6.49	1.09	7.36	1.62
AB5	1.81	0.76	1.22	0.72	7.54	2.03	7.34	1.31	7.92	1.62
**PM_2.5_ concentration (µg/m^3^)**										
DustTrak	4.78	0.63	6.72	2.34	16.52	2.12	13.67	1.27	16.51	2.47
AB1	3.06	1.04	2.82	1.11	11.21	2.63	11.16	1.71	11.04	1.85
AB2	2.85	0.99	2.57	0.98	11.74	2.51	11.39	1.82	11.39	1.82
AB3	2.47	0.98	1.97	0.91	10.01	2.28	9.63	1.56	9.94	1.58
AB4	2.81	0.99	2.61	1.05	10.36	2.44	10.04	1.38	10.81	1.69
AB5	3.36	1.01	2.75	1.03	10.93	2.46	11.04	1.72	11.38	1.67
**PM_10_ concentration (µg/m^3^)**										
DustTrak	4.89	0.64	7.91	3.35	19.01	2.43	14.48	1.41	18.76	3.05
AB1	3.51	1.13	3.55	1.38	15.31	4.21	14.89	2.91	15.17	3.15
AB2	3.18	1.01	3.26	1.28	16.42	4.13	15.45	3.22	15.91	3.13
AB3	2.74	1.01	2.45	1.01	12.98	3.33	12.18	2.32	12.91	2.57
AB4	3.17	1.07	3.26	1.26	13.76	3.67	13.16	2.39	14.42	2.86
AB5	3.84	1.09	3.57	1.33	14.67	3.81	14.32	2.79	15.42	2.87

**Table 3:** Evaluation of 1 min PM_1_, PM_2.5_, and PM_10_ average concentrations (µg/m^3^) reported by AirBeam2 sensors compared with the DustTrak sensor in different environments.

Sensors	Linear Regression	R^2^	%Bias	Linear Regression	R^2^	%Bias	Linear Regression	R^2^	%Bias
Office (Indoor)		PM_1_		PM_2.5_			PM_10_		
AB1	y = 0.67x + 3.66	0.72	397	y = 0.52x + 3.16	0.72	71	y = 0.45x + 3.27	0.65	51
AB2	y = 0.69x + 3.71	0.76	469	y = 0.57x + 3.13	0.78	85	y = 0.52x + 3.21	0.69	66
AB3	y = 0.69x + 3.88	0.71	661	y = 0.57x + 3.36	0.76	122	y = 0.53x + 3.39	0.73	101
AB4	y = 0.66x + 3.79	0.76	567	y = 0.56x = 3.19	0.76	88	y = 0.48x + 3.33	0.68	69
AB5	y = 0.73x + 3.39	0.77	222	y = 0.54x + 2.92	0.76	51	y = 0.48x + 3.03	0.64	34
**Office (Outdoor)**		**PM_1_**		**PM_2.5_**			**PM_10_**		
AB1	y = 5.53x − 0.73	0.11	682	y = 3.42x − 1.84	0.17	184	y = 7.84x − 17.44	0.24	182
AB2	y = 6.36x − 0.67	0.12	776	y = 3.82x − 2.03	0.17	222	y = 7.01x − 12.29	0.16	227
AB3	y = 8.07x − 1.47	0.16	1387	y = 4.64x − 1.44	0.23	363	y = 10.70x − 16.34	0.32	327
AB4	y = 5.64x − 1.28	0.11	741	y = 3.72x − 1.94	0.19	235	y = 8.01x − 15.64	0.21	218
AB5	y = 6.51x − 0.31	0.13	651	y = 3.51x − 1.83	0.16	197	y = 7.05x − 14.69	0.18	189
**MTR station(Platform)**		**PM_1_**		**PM_2.5_**			**PM_10_**		
AB1	y = 0.84x + 9.41	0.76	173	y = 0.69x + 8.72	0.75	52	y = 0.47x + 11.87	0.65	31
AB2	y = 0.87x + 8.63	0.76	98	y = 0.74x + 7.81	0.77	43	y = 0.47x + 11.21	0.65	21
AB3	y = 0.95x + 9.21	0.78	139	y = 0.82x + 8.29	0.78	69	y = 0.59x + 11.25	0.67	53
AB4	y = 0.94x + 9.25	0.77	137	y = 0.76x + 8.65	0.76	64	y = 0.53x + 11.71	0.64	45
AB5	y = 0.87x + 9.31	0.72	121	y = 0.73x + 8.57	0.71	55	y = 0.48x + 11.94	0.57	35
**MTR station(Lobby)**		**PM_1_**		**PM_2.5_**			**PM_10_**		
AB1	y = 0.78x + 7.76	0.71	88	y = 0.65x + 6.42	0.76	23	y = 0.41x + 8.45	0.69	−1
AB2	y = 0.73x + 7.86	0.68	81	y = 0.59x + 6.89	0.72	21	y = 0.35x + 9.01	0.65	−3
AB3	y = 0.81x + 8.31	0.69	115	y = 0.69x + 7.03	0.72	43	y = 0.51x + 8.37	0.68	21
AB4	y = 0.89x + 7.64	0.64	110	y = 0.74x + 6.23	0.65	37	y = 0.46x + 8.42	0.61	12
AB5	y = 0.77x + 7.78	0.67	86	y = 0.63x + 6.74	0.72	25	y = 0.41x + 8.69	0.64	3
**Seaside**		**PM_1_**		**PM_2.5_**			**PM_10_**		
AB1	y = 0.66x + 10.93	0.19	117	y = 0.61x + 9.79	0.21	51	y = 0.44x + 11.95	0.22	27
AB2	y = 0.65x + 10.71	0.21	103	y = 0.66x + 8.94	0.24	46	y = 0.47x + 11.27	0.23	20
AB3	y = 0.66x + 11.37	0.16	137	y = 0.67x + 9.79	0.19	68	y = 0.53x + 11.86	0.21	48
AB4	y = 0.64x + 11.17	0.17	121	y = 0.64x + 9.61	0.19	54	y = 0.46x + 12.14	0.18	33
AB5	y = 0.63x + 10.92	0.17	105	y = 0.64x + 9.21	0.19	47	y = 0.46x + 11.74	0.18	24

**Table 4:** Summary of cross-validation R^2^, ME, RMSE, and bias percentage for calibration models based on data collected in all selected environments.

Include Data Collected in the Office (Outdoor)								
Sensors	R^2^	ME (µg/m^3^)	RMSE (µg/m^3^)	% Bias	R^2^	ME (µg/m^3^)	RMSE (µg/m^3^)	% Bias
	MLR Models				RF Models			
**PM_1_**								
AB1	0.51	1.45	4.64	−0.57	0.59	1.19	4.26	−2.54
AB2	0.52	1.34	4.58	−0.42	0.47	1.79	4.81	−0.33
AB3	0.63	1.32	3.67	−0.68	0.59	1.65	3.85	−0.04
AB4	0.51	1.51	4.66	−0.46	0.47	1.78	4.63	−0.12
AB5	0.50	1.53	4.67	−0.91	0.46	1.79	4.87	−0.21
**PM_2.5_**								
AB1	0.44	1.53	5.61	0.22	0.41	1.99	5.76	−0.13
AB2	0.44	1.42	5.61	0.32	0.40	2.02	5.82	−0.32
AB3	0.55	1.33	4.47	0.15	0.51	1.87	4.70	−0.02
AB4	0.54	1.43	4.54	0.33	0.52	1.83	4.69	−0.09
AB5	0.43	1.59	5.65	−0.03	0.38	2.06	5.87	−0.38
**PM_10_**								
AB1	0.18	2.68	12.88	1.12	0.22	3.01	12.50	−0.94
AB2	0.17	2.50	12.92	0.90	0.16	3.00	13.02	−0.15
AB3	0.27	2.18	9.82	1.23	0.41	2.67	8.84	−0.13
AB4	0.25	2.26	9.98	1.11	0.26	2.73	11.49	−0.22
AB5	0.21	2.46	11.11	0.10	0.21	2.84	11.11	−0.29

**Table 5:** Evaluation of 1 min PM_1_, PM_2.5_, and PM_10_ average concentrations (µg/m^3^) reported by AirBeam2 sensors compared with the DustTrak sensor in different environments.

Exclude Data Collected in the Office (Outdoor)								
Sensors	R^2^	ME (µg/m^3^)	RMSE (µg/m^3^)	% Bias	R^2^	ME (µg/m^3^)	RMSE (µg/m^3^)	% Bias
	MLR Models				RF Models			
**PM_1_**								
AB1	0.91	1.02	1.49	−1.00	0.94	0.72	1.15	−0.08
AB2	0.92	0.90	1.36	−0.73	0.92	1.02	1.42	−0.16
AB3	0.90	1.03	1.52	−1.07	0.94	0.70	1.17	−0.04
AB4	0.89	1.10	1.59	−0.85	0.95	0.69	1.13	−0.05
AB5	0.89	1.16	1.66	−1.21	0.90	1.15	1.56	−0.05
**PM_2.5_**								
AB1	0.93	0.93	1.39	−0.64	0.95	0.74	1.18	−0.08
AB2	0.94	0.81	1.26	−0.30	0.94	0.76	1.23	−0.05
AB3	0.93	0.90	1.36	−0.57	0.94	0.74	1.20	−0.09
AB4	0.92	1.01	1.46	−0.43	0.93	0.93	1.34	−0.09
AB5	0.91	1.04	1.50	−0.73	0.95	0.74	1.18	−0.07
**PM_10_**								
AB1	0.92	1.24	1.75	−0.98	0.94	0.96	1.47	−0.10
AB2	0.93	1.09	1.59	−0.51	0.94	1.01	1.53	−1.13
AB3	0.92	1.16	1.69	−0.91	0.94	0.94	1.46	−0.10
AB4	0.91	1.30	1.80	−0.66	0.93	1.06	1.58	−0.66
AB5	0.90	1.43	1.95	−1.29	0.94	0.97	1.49	−0.03

## Data Availability

The datasets generated from the current study are available from the corresponding author on reasonable request.

## Supplementary Materials

The following supporting information can be downloaded at: https://www.mdpi.com/article/10.3390/s22062381/s1, Figure S1: Correlations for the PM_1_, PM_2.5_, and PM_10_ concentrations (µg/m^3^) reported by the AirBeam2 sensors in different environments and aggregated in different temporal units (i.e., 5-sec, 1-min, 10-min and 30-min); Figure S2: R^2^ of Linear regression models for the PM_1_, PM_2.5_, and PM_10_ concentrations (µg/m^3^) reported by AirBeam2 and DustTrak sensors, and aggregated in different temporal units (i.e., 5-sec, 1-min, 10-min and 30-min); Figure S3: Bias percentage for the PM_1_, PM_2.5_, and PM_10_ concentrations (µg/m^3^) reported by AirBeam2 and DustTrak sensors and aggregated in different temporal units (i.e., 5-sec, 1-min, 10-min and 30-min).
