# Supplementary material for: Field Evaluation and Calibration of Low-Cost Air Pollution Sensors for Environmental Exposure Research
Source: Sensors (Basel). 2022 Mar 19;22(6):2381. doi: 10.3390/s22062381 (PMC8948698; doi:10.3390/s22062381)
Supplement: Supplementary file 1 [file sensors-22-02381-s001.zip › sensors-1635113-supplementary.pdf]

Supporting Information

Field Evaluation and Calibration of Low-Cost Air Pollution Sensors for Environmental Exposure Research

Jianwei Huang[1], Mei-Po Kwan[1,2,*], Jiannan Cai[1], Wanying Song[1], Changda Yu[1], Zihan Kan[1], Steve Hung-Lam Yim[3,4,5]

[1] Institute of Space and Earth Information Science, The Chinese University of Hong Kong, Hong Kong, China.

[2] Department of Geography and Resource Management, The Chinese University of Hong Kong, Hong Kong, China.

[3] Asian School of the Environment, Nanyang Technological University, Singapore.

[4] Lee Kong Chian School of Medicine, Nanyang Technological University, Singapore.

[5] Earth Observatory of Singapore, Nanyang Technological University, Singapore.

* Corresponding author

Email: mpkwan@cuhk.edu.hk

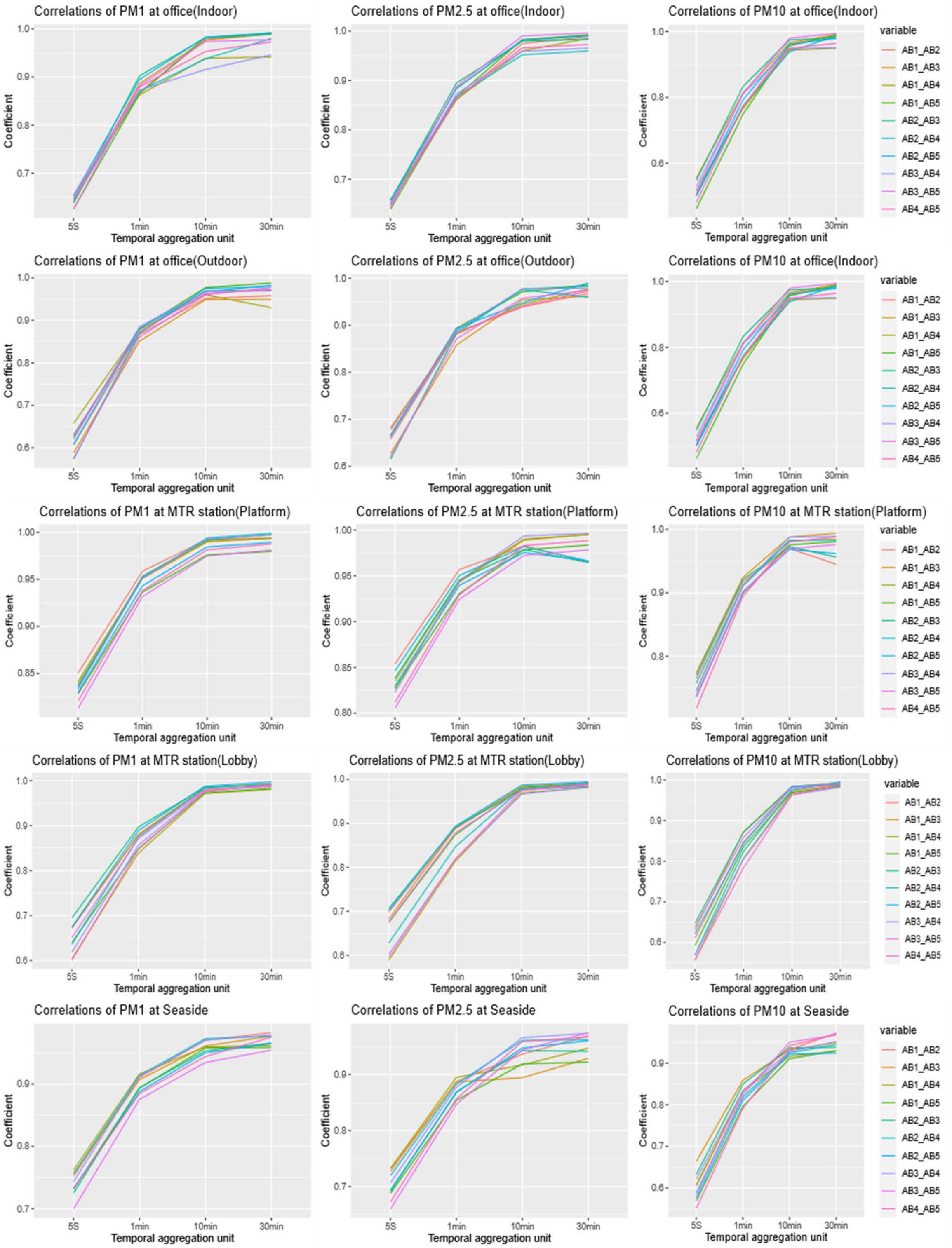

Figure S1. Correlations for the PM_1, $PM_{2.5}$, and PM_{10} concentrations (μg/m^3) reported by the AirBeam2 sensors in different environments and aggregated in different temporal units (i.e., 5-sec, 1-min, 10-min and 30-min).

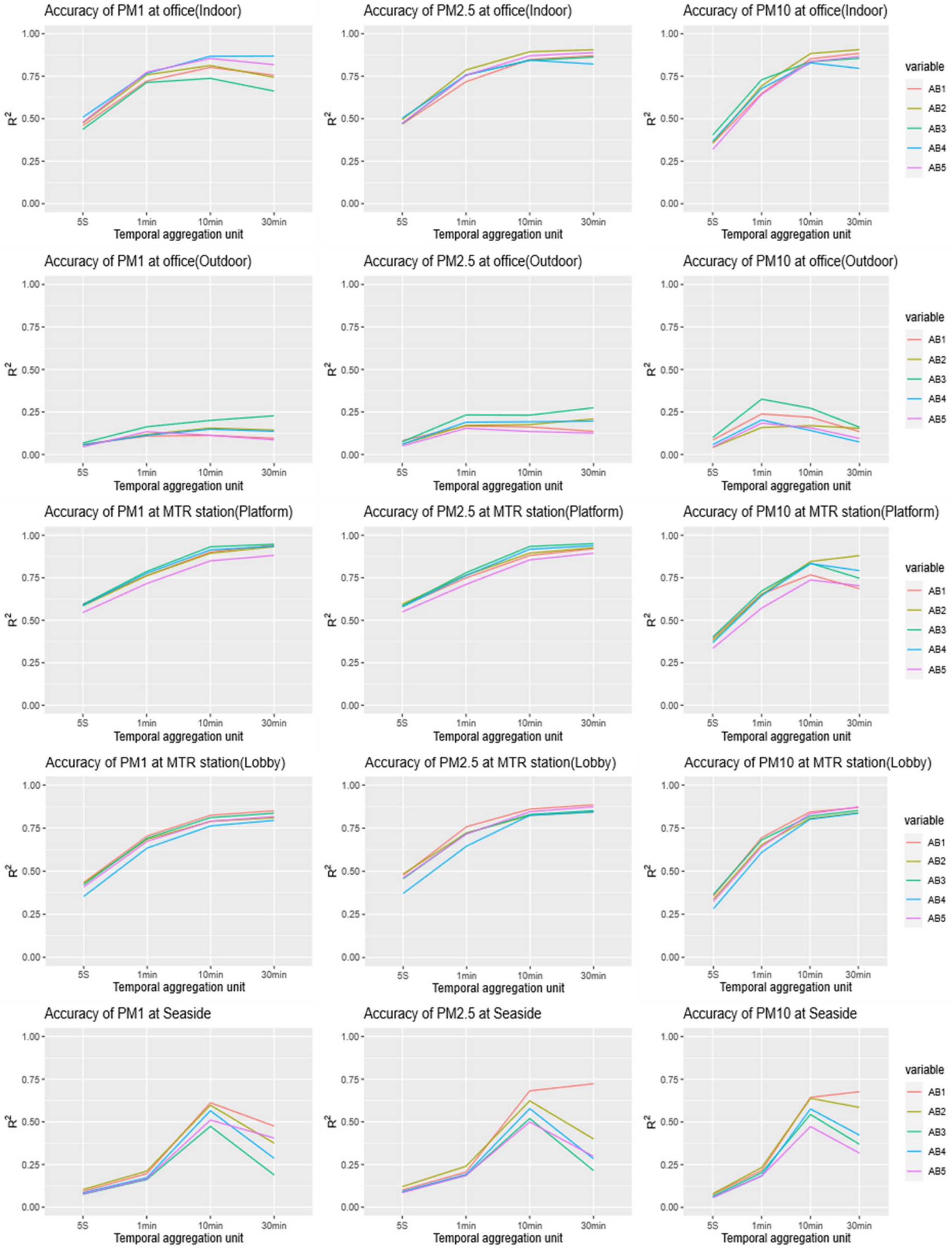

Figure S2. R^2 of Linear regression models for the PM_1, $PM_{2.5}$, and PM_{10} concentrations (μg/m^3) reported by AirBeam2 and DustTrak sensors, and aggregated in different temporal units (i.e., 5-sec, 1-min, 10-min and 30-min).

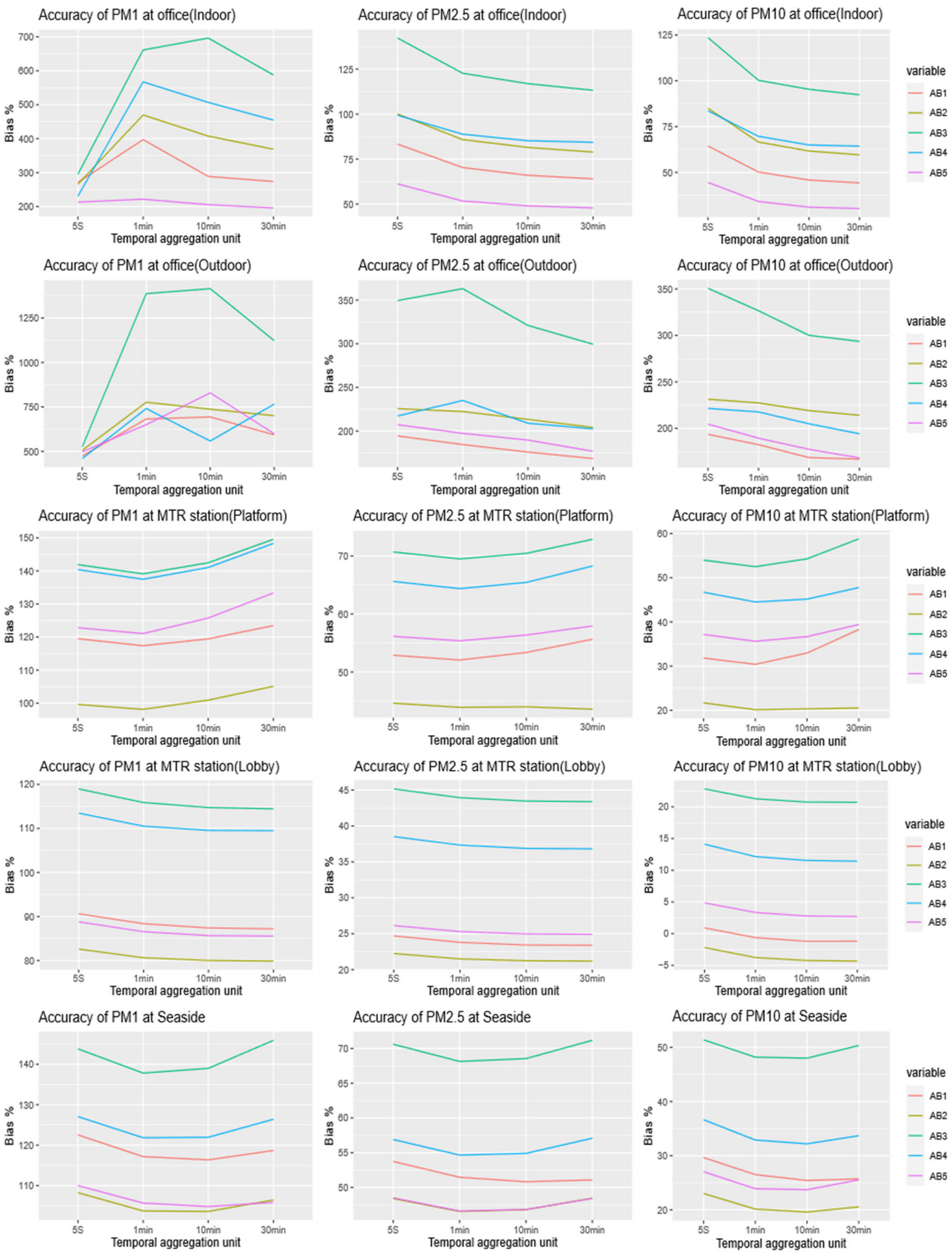

Figure S3. Bias percentage for the PM1, PM2.5, and PM10 concentrations (μg/m^3) reported by AirBeam2 and DustTrak sensors and aggregated in different temporal units (i.e., 5-sec, 1-min, 10-min and 30-min).
